# Stereotactic radiosurgery for brain metastases: evolving practice patterns from the national cancer database (2004–2020)

**DOI:** 10.1007/s11060-025-05178-8

**Published:** 2025-08-22

**Authors:** Jonathan J. Shih, Bhav Jain, Rohan Patel, Urvish Jain, Miranda Lam, Fumiko Chino, Manali I. Patel, Edward Christopher Dee, Erqi Pollom, Gordon Li, Kekoa Taparra

**Affiliations:** 1 School of Medicine, University of California, San Francisco, San Francisco, CA USA; 2 School of Medicine, Stanford University, Stanford, CA USA; 3 Department of Radiation Oncology, University Hospitals Seidman Cancer Center, Case Western Reserve University, Cleveland, OH USA; 4 School of Medicine, University of Pittsburgh, Pittsburgh, PA USA; 5 Department of Radiation Oncology, Dana Farber Cancer Institute, Brigham and Women’s Hospital, Harvard Medical School, Boston, MA USA; 6 Department of Health Policy and Management, Harvard T. H. Chan School of Public Health, Boston, MA USA; 7 Department of Radiation Oncology, MD Anderson Cancer Center, Houston, TX USA; 8 Department of Medicine, Stanford Medicine, Stanford, CA USA; 9 Medical Services, VA Palo Alto Health Care System, Palo Alto, CA USA; 10 Department of Radiation Oncology, Memorial Sloan Kettering Cancer Center, New York, NY USA; 11 Department of Radiation Oncology, Stanford Medicine, Stanford, CA USA; 12 Department of Neurosurgery, Stanford Medical, Palo Alto, CA USA; 13 Department of Health Policy and Management, Fielding School of Public Health, University of California, Los Angeles, CA USA; 14 Department of Radiation Oncology, David Geffen School of Medicine, University of California, Los Angeles, Los Angeles, CA USA

**Keywords:** Stereotactic radiosurgery, Whole brain radiation therapy, Brain Neoplasms / secondary, Radiotherapy / trends, Healthcare disparities, Socioeconomic factors

## Abstract

**Purpose:**

Stereotactic radiosurgery (SRS) offers less neurotoxicity and comparable survival to whole-brain radiation therapy (WBRT) for brain metastases (BM). Current SRS practice patterns are understudied. We examined national trends in SRS and WBRT utilization.

**Methods:**

We queried the National Cancer Database for patients with BM from twelve cancers (≥ 18 years; diagnosed 2004–2020) treated with radiotherapy. Patients were grouped by first-course radiotherapy modality (SRS:1–5 fractions; WBRT:5–15 fractions). Multivariable logistic regression assessed SRS predictors, adjusting for sociodemographic and clinical variables. A race*diagnosis year interaction evaluated temporal trends. Difference-in-differences analysis assessed Medicaid expansion impact.

**Results:**

Of 89,984 patients, 24,174 (27%) received SRS. SRS utilization rose from 8 to 54% between 2004 and 2020 (*P* < 0.001). SRS was more likely in patients diagnosed in recent years (aOR = 3.85 [95% CI = 3.70–4.01]), who received prior chemotherapy (aOR = 1.17 [1.13–1.21]) or surgery (aOR = 2.25 [2.11–2.40]), and those with colorectal (aOR = 1.93 [1.64–2.26]), lung (aOR = 1.37 [1.24–1.50]), melanoma (aOR = 2.76 [2.46–3.10]), thyroid (aOR = 2.17 [1.36–3.46]), or kidney/bladder cancer (aOR = 2.76 [2.44–3.12]) versus breast cancer. SRS was less likely in patients with lower income (aOR = 0.88 [0.85–0.92]) or educational attainment (aOR = 0.88 [0.85–0.92]), Medicaid/Medicare (aOR = 0.86 [0.83–0.90]), no insurance (aOR = 0.49 [0.44–0.53]), or treatment at community (aOR = 0.31 [0.29–0.34]), comprehensive community (aOR = 0.56 [0.54–0.58]), or integrated facilities (aOR = 0.77 [0.73–0.80]). Race and ethnicity were not overall associated with SRS use. Medicaid expansion had no impact (aOR = 0.99 [0.88–1.10]).

**Conclusions:**

SRS utilization increased between 2004 and 2020. However, disparities persist for patients with lower socioeconomic status, uninsured/public insurance, or non-academic center treatment, potentially reflecting access disparities or differences in disease burden. Targeted efforts are needed to ensure equitable access to advanced cancer therapies, particularly in the context of potential additive effects of disparities in disease burden.

**Supplementary Information:**

The online version contains supplementary material available at 10.1007/s11060-025-05178-8.

## Introduction

Each year, more than 200,000 patients with cancer in the United States develop brain metastases (BM) [1]. BM are associated with high mortality and morbidity, including severe neurological symptoms that profoundly impact quality of life [2]. Whole brain radiation therapy (WBRT) has traditionally been used as a primary treatment modality for patients with BM due to its efficacy in managing extensive disease at the expense of higher neurocognitive toxicity [3]. Stereotactic radiosurgery (SRS) emerged in the late 1990s as an accepted alternative treatment for limited BM and select cases of extensive disease [4, 5]. Beginning in 2016, studies have suggested that SRS offers comparable survival outcomes than WBRT with a reduced risk of neurotoxicity, making it an increasing preferred treatment modality [6, 7].

Disparities in SRS utilization exist, however, and are due in part to limited access to academic centers with SRS expertise, socioeconomic barriers such as lower income and insurance coverage, and systemic inequities affecting minoritized populations. Patients from marginalized backgrounds are more likely to present with advanced disease requiring WBRT, face treatment delays, and lack access to specialized care [8–10]. Earlier data suggest that patients from racially and ethnically marginalized communities, those without private insurance, and those residing in lower-income or less-educated regions were less likely to receive SRS [7]. However, no studies have fully disaggregated all racial categories, which is critical given studies on data disaggregation have highlighted that heterogeneity within racial and ethnic groups is often overlooked in cancer research [11–13]. Furthermore, recent trends in SRS utilization amid an evolving United States healthcare landscape have not been explored. For instance, Medicaid expansion under the 2010 Affordable Care Act aimed to increase healthcare access for low-income populations, possibly improving access to advanced cancer treatments [14].

Given these considerations, SRS utilization patterns in the United States may have shifted over the past decade, with prior aggregated analyses potentially concealing disparities in access to treatment of BM. In this study, we define disparities as significant differences in treatment utilization (SRS versus WBRT) associated with sociodemographic factors (race and ethnicity, socioeconomic status, and insurance coverage), geographic location, or healthcare access (availability of Medicaid expansion and institutional factors). Using updated National Cancer Database (NCDB) data for patients treated for BM through 2020, we aimed to evaluate trends in SRS versus WBRT utilization and assess whether disparities in SRS utilization have changed over time. By incorporating a broader range of primary cancer sites, disaggregated racial and ethnic data, and Medicaid expansion status, we sought to provide a more comprehensive understanding of the factors influencing treatment patterns for BM.

## Methods

### Data source and study population

The NCDB, a joint project of the Commissions on Cancer of the American College of Surgeons and the American Cancer Society, is a comprehensive hospital-based dataset that captures clinical oncology data from over 1,500 accredited facilities. This retrospective cohort study utilized data derived from NCDB, which includes more than 70% of all newly diagnosed malignancies in the United States [15]. The NCDB was queried for all adult patients (≥ 18 years old) treated with radiotherapy (RT) for a BM diagnosis between 2004 and 2020 and with known follow-up. Twelve cancers were included based on their high prevalence in the United States to ensure sufficient sample size for analysis (breast, colorectal, kidney/bladder, liver, lung, lymphoma, and melanoma). Additional common cancers with lower central nervous system tropism (endometrial, oral cavity, pancreas, prostate, and thyroid) were included for a more comprehensive analysis and statistical robustness [16]. BM was defined using the corresponding variable “METS_AT_DX_BRAIN” coded in the NCDB [15].

Patients were grouped based on the RT modality received as part of their first course of treatment, categorized as SRS or WBRT. RT modality, fractionation, and target are explicitly coded in the NCDB and were used for classification. The SRS cohort was defined as patients who received stereotactic RT or radiosurgery (not otherwise specified), robotic stereotactic RT or radiosurgery (e.g., CyberKnife), or Gamma knife radiosurgery, with treatment administered over 1–5 fractions. The WBRT cohort was defined as patients who received external beam (not otherwise specified) RT, conformal or 3-D conformal RT, or intensity-modulated RT, with treatment administered over 5–15 fractions. Definitions of SRS and WBRT are consistent with National Comprehensive Cancer Network guidelines and capture the standard fractionation patterns [17]. Patients were excluded if: RT modality or fractionation was unknown, first phase of RT received was not for the brain, or data were missing for any model covariates (listed below).

This study was reviewed and approved as exempt from full review by the Stanford University Institutional Review Board because de-identified and publicly available data in the NCDB do not constitute human subjects research.

### Clinical and sociodemographic covariates

The primary dependent variable of interest was receipt of SRS versus WBRT. Independent variables included race, ethnicity, sex, age, year of diagnosis (categorized as 2004–2011 vs. 2012–2020 to reflect the midpoint of the study period), distance to hospital, household income (median income of households in patient’s ZIP code), rurality, educational attainment (percentage of residents in patient’s ZIP code without a high school diploma), insurance status, Charlson-Deyo comorbidity score, geographical region of hospital, facility type, cancer type, receipt of chemotherapy, and receipt of surgery on the primary site. Higher versus lower income and educational attainment were defined as above or below the median, respectively. Patient data corresponding to income and education were categorized into quartiles that were derived from the 2012 American Community Survey based on the patient’s ZIP code.

Race included: White, Black, East Asian (Chinese, Japanese, or Korean), South Asian (Asian Indian or Pakistani), Southeast Asian (Filipino, Vietnamese, Laotian, Hmong, Kampuchean, or Thai), American Indian or Alaskan Native, and Native Hawaiian or Pacific Islander (Native Hawaiian, Micronesian, Chamoru/Guamanian, Tahitian, Sāmoan, Tongan, Melanesian, Fiji Islander, New Guinean, Other Polynesian, or Pacific Islander Not Otherwise Specified).

### Statistical analysis

Baseline demographic statistics were analyzed to compare dependent and independent variables by race and ethnicity and treatment cohort. Graphical assessments were used to evaluate the proportion of RT attributed to SRS versus WBRT over time. Univariable and multivariable logistic regressions were performed to examine associations between independent variables and receipt of SRS versus WBRT. Odds ratios (ORs) and adjusted odds ratios (aORs) with 95% confidence intervals (95%CI) were reported, where an OR or aOR > 1 indicated greater odds of being treated with SRS. The multivariable model adjusted for all independent variables. Disparities in treatment were specifically assessed by examining associations between treatment receipt and sociodemographic characteristics, insurance status, and institutional factors. To assess temporal trends in racial and ethnic disparities in receipt of SRS, a multivariable logistic regression model was constructed with an interaction term between race and ethnicity and year of diagnosis.

To assess the effect of Medicaid expansion on SRS versus WBRT utilization, a difference-in-differences analysis was conducted to control for temporal trends unrelated to expansion status, consistent with prior research [18]. Given that 24 states and the District of Columbia expanded their Medicaid programs by January 1, 2014, this date marked the beginning of the post-expansion period in our sub-analysis [19]. The sub-analysis was restricted to patients aged 40–64, as Medicaid expansion status was unavailable for patients younger than 40 years, and patients 65 or older are eligible for Medicare. A multivariable logistic regression model was constructed using maximum likelihood estimation to obtain aORs and 95%CIs. The model included all independent variables, as well as residence in a Medicaid expansion state, diagnosis in the post-expansion period (during or after 2014), and an interaction term between these two factors. To prevent multicollinearity, the original year of diagnosis variable was excluded. All analyses were performed with R version 4.3.1 [20].

## Results

### Patient characteristics and overall trends of SRS use

**Fig. 1:**
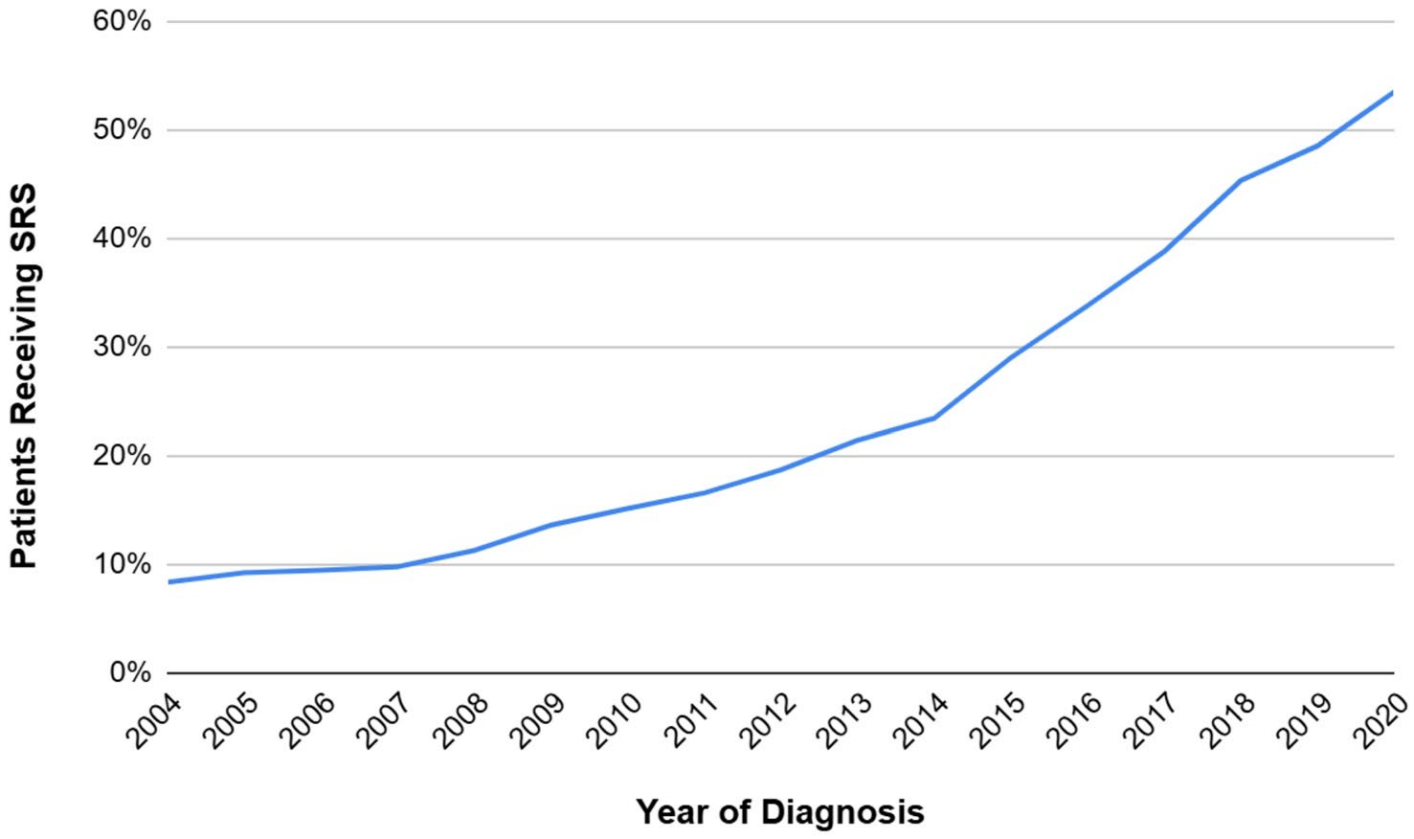
Temporal trends in stereotactic radiosurgery utilization for patients in the United States diagnosed with brain metastases between 2004–2020

Among 160,286 adult patients (≥ 18 years old) treated with RT for BM between 2004 and 2020 with known follow-up, 89,984 patients remained after applying exclusionary criteria (Appendix 1), of whom 24,174 (27%) received SRS and 65,810 (73%) received WBRT. SRS utilization increased significantly from 8% in 2004 to 54% in 2020 (*P* < 0.001) with an average annual increase of 2.8% (Fig. 1). The average annual increase of SRS uptake was higher from 2012 to 2020 compared to 2004–2011 (4.4 vs. 1.3% per year, respectively). Characteristics of the study cohort by RT modality are listed in Table 1. The median age was 64 years (IQR 57–72), and most common primary cancer was lung (86%). Overall, most patients were White (86%), diagnosed in 2012–2020 (64%), had higher income (≥$48,000) (57%), had higher educational attainment (≥ 87.1% of residents in patient’s ZIP code with high school diploma) (55%), and publicly insured with Medicare/Medicaid (60%). Most patients underwent chemotherapy (63%) prior to RT, did not have surgery for their primary tumor (94%), and had a Charlson-Deyo comorbidity score of 2 lower (96%). Characteristics of the study cohort by race are listed in Appendix 2.

**Table 1:** Characteristics of patients with brain metastases treated with radiotherapy from 2004 to 2020

Characteristic	Overall (*N* = 89,984)	WBRT^a^ (*N* = 65,810)	SRS^b^ (*N* = 24,174)	*P*-value
**Race**,** n (%)**				**< 0.001**
White	77,556 (86)	56,646 (86)	20,910 (86)	
American Indian or Alaskan Native	296 (0.3)	209 (0.3)	87 (0.4)	
Black	10,028 (11)	7,498 (11)	2,530 (10)	
East Asian	912 (1.0)	632 (1.0)	280 (1.2)	
Native Hawaiian or Pacific Islander	168 (0.2)	115 (0.2)	53 (0.2)	
South Asian	250 (0.3)	158 (0.2)	92 (0.4)	
Southeast Asian	774 (0.9)	552 (0.8)	222 (0.9)	
**Ethnicity**,** n (%)**				0.073
Non-Hispanic	87,186 (97%)	63,805 (97%)	23,381 (97%)	
Hispanic	2,798 (3.1)	2,005 (3.0)	793 (3.3)	
**Sex**,** n (%)**				0.50
Male	46,296 (51%)	33,901 (52%)	12,395 (51%)	
Female	43,688 (49%)	31,909 (48%)	11,779 (49%)	
**Age**,** Median (IQR)**^**c**^	64 (57–72)	64 (57–71)	65 (58–72)	**< 0.001**
**Year Diagnosis**,** n (%)**				**< 0.001**
2004–2011	31,975 (36)	28,030 (43)	3,945 (16)	
2012–2020	58,009 (64)	37,780 (57)	20,229 (84)	
**Distance To Hospital**,** Median (IQR)**	10 (4–23)	9 (4–21)	13 (6–30)	**< 0.001**
**Income**,** n (%)**				**< 0.001**
Higher Income	50,860 (57)	36,130 (55)	14,730 (61)	
Lower Income	39,124 (43)	29,680 (45)	9,444 (39)	
**Rurality**,** n (%)**				**< 0.001**
Metropolitan	74,092 (82)	53,844 (82)	20,248 (84)	
Urban-Rural	15,892 (18)	11,966 (18)	3,926 (16)	
**Education**,** n (%)**				**< 0.001**
More Education	49,116 (55)	34,965 (53)	14,151 (59)	
Less Education	40,868 (45)	30,845 (47)	10,023 (41)	
**Insurance Status**,** n (%)**				**< 0.001**
Private Insurance	31,713 (35)	22,813 (35)	8,900 (37)	
Medicaid/Medicare	54,106 (60)	39,462 (60)	14,644 (61)	
Uninsured	4,165 (4.6)	3,535 (5.4)	630 (2.6)	
**Comorbidity Index**,** n (%)**				**< 0.001**
0	59,409 (66)	43,026 (65)	16,383 (68)	
1	20,008 (22)	15,100 (23)	4,908 (20)	
2	6,916 (7.7)	5,119 (7.8)	1,797 (7.4)	
3+	3,651 (4.1)	2,565 (3.9)	1,086 (4.5)	
**US Region**,** n (%)**				**< 0.001**
Northeast	19,022 (21)	13,288 (20)	5,734 (24)	
Midwest	26,326 (29)	20,019 (30)	6,307 (26)	
South	31,492 (35)	23,071 (35)	8,421 (35)	
West	13,144 (15)	9,432 (14)	3,712 (15)	
**Facility Type**,** n (%)**				**< 0.001**
Academic	29,943 (33)	19,493 (30)	10,450 (43)	
Community	6,452 (7.2)	5,596 (8.5)	856 (3.5)	
Comp. Community	35,843 (40)	27,844 (42)	7,999 (33)	
Integrated	17,746 (20)	12,877 (20)	4,869 (20)	
**Cancer Type**,** n (%)**				**< 0.001**
Breast	2,803 (3.1)	2,175 (3.3)	628 (2.6)	
Colorectal	1,100 (1.2)	691 (1.0)	409 (1.7)	
Endometrial	272 (0.3)	180 (0.3)	92 (0.4)	
Kidney/Bladder	3,100 (3.4)	1,709 (2.6)	1,391 (5.8)	
Liver	77 (< 0.1)	53 (< 0.1)	24 (< 0.1)	
Lung	77,406 (86)	57,816 (88)	19,590 (81)	
Lymphoma	426 (0.5)	402 (0.6)	24 (< 0.1)	
Melanoma	4,232 (4.7)	2,371 (3.6)	1,861 (7.7)	
Oral Cavity	42 (< 0.1)	35 (< 0.1)	< 11^d^ (< 0.1)	
Pancreas	266 (0.3)	200 (0.3)	66 (0.3)	
Prostate	173 (0.2)	131 (0.2)	42 (0.2)	
Thyroid	87 (< 0.1)	47 (< 0.1)	40 (0.2)	
**Chemotherapy**,** n (%)**	56,713 (63)	41,358 (63)	15,355 (64)	0.063
**Surgery Status**,** n (%)**				**< 0.001**
No Surgery Performed	84,861 (94)	62,961 (96)	21,900 (91)	
Surgery Performed	5,123 (5.7)	2,849 (4.3)	2,274 (9.4)	

^*a*^ *WBRT = Whole Brain Radiation Therapy*

^*b*^ *SRS = Stereotactic Radiosurgery*

^*c*^ *IQR = Interquartile Range*

^*d*^ *n < 11 masked per NCDB data privacy policy*

### Factors associated with SRS use

Factors associated with SRS use on univariable and multivariable analysis are shown in Table 2. On multivariable analysis, patients were more likely to be treated with SRS if they were female, diagnosed in more recent years (2012–2020 vs. 2004–2011), had previously received chemotherapy, had previously received surgery, and had primary colorectal, endometrial, kidney, bladder, lung, melanoma, and thyroid cancer compared to the majority breast cancer group. Patients were less likely to be treated with SRS if they had lower income, had lower educational attainment, were uninsured or had public (Medicaid/Medicare) insurance, had a comorbidity index of 1 compared to 0, were located in the Midwest compared to the Northeast, were treated at non-academic medical centers, and had primary lymphoma compared to primary breast cancer. South Asian race was associated with increased SRS use; however, the difference was marginally significant (*p* = 0.045). Race and ethnicity were otherwise not significantly associated with receiving SRS. In the interaction term analysis, no significant temporal trends in SRS use were observed for any racial groups.

**Table 2:** Factors associated with receipt of stereotactic radiosurgery vs. Whole-Brain radiation therapy

	Univariable		Multivariable	
**Characteristic**	**OR (95%CI)** ^*a*^	***P*** ** value**	**aOR (95%CI)** ^*b*^	***P*** ** value**
**Race**				
White	—		—	
American Indian or Alaskan Native	1.13 (0.87–1.44)	0.347	1.21 (0.53–2.43)	0.62
Black	0.91 (0.87–0.96)	**< 0.001**	0.91 (0.81–1.02)	0.12
East Asian	1.20 (1.04–1.38)	**0.012**	0.93 (0.65–1.29)	0.67
Native Hawaiian or Pacific Islander	1.25 (0.89–1.72)	0.182	1.66 (0.83–3.09)	0.13
South Asian	1.58 (1.22–2.04)	**< 0.001**	1.79 (0.98–3.10)	**0.045**
Southeast Asian	1.09 (0.93–1.27)	0.283	0.87 (0.56–1.29)	0.51
**Ethnicity**				
Non-Hispanic	—		—	
Hispanic	1.08 (0.99–1.17)	0.073	1.01 (0.92–1.10)	0.84
**Sex**				
Male	—		—	
Female	1.01 (0.98–1.04)	0.524	1.06 (1.03–1.10)	**< 0.001**
**Age**	1.01 (1.01–1.01)	**< 0.001**	1.01 (1.01–1.01)	**< 0.001**
**Year Diagnosis**				
2004–2011	—		—	
2012–2020	3.80 (3.66–3.95)	**< 0.001**	3.85 (3.70–4.01)	**< 0.001**
**Distance To Hospital**	1.00 (1.00–1.00)	**< 0.001**	1.00 (1.00–1.00)	**< 0.001**
**Income**				
Higher Income	—		—	
Lower Income	0.78 (0.76–0.80)	**< 0.001**	0.88 (0.85–0.92)	**< 0.001**
**Rurality**				
Metropolitan	—		—	
Urban-Rural	0.87 (0.84–0.91)	**< 0.001**	1.02 (0.97–1.06)	0.51
**Education**				
More Education	—		—	
Less Education	0.80 (0.78–0.83)	**< 0.001**	0.88 (0.85–0.92)	**< 0.001**
**Insurance Status**				
Private Insurance	—		—	
Medicaid/Medicare	0.95 (0.92–0.98)	**0.002**	0.86 (0.83–0.90)	**< 0.001**
Uninsured	0.46 (0.42–0.50)	**< 0.001**	0.49 (0.44–0.53)	**< 0.001**
**Comorbidity Index**				
0	—		—	
1	0.85 (0.82–0.89)	**< 0.001**	0.89 (0.86–0.93)	**< 0.001**
2	0.92 (0.87–0.98)	**0.005**	0.94 (0.89–1.00)	0.064
3+	1.11 (1.03–1.20)	**0.004**	1.07 (0.99–1.16)	0.081
**US Region**				
Northeast	—		—	
Midwest	0.73 (0.70–0.76)	**< 0.001**	0.79 (0.75–0.83)	**< 0.001**
South	0.85 (0.81–0.88)	**< 0.001**	0.98 (0.94–1.03)	0.46
West	0.91 (0.87–0.96)	**< 0.001**	1.01 (0.96–1.07)	0.64
**Facility Type**				
Academic	—		—	
Community	0.29 (0.26–0.31)	**< 0.001**	0.31 (0.29–0.34)	**< 0.001**
Comprehensive Community	0.54 (0.52–0.55)	**< 0.001**	0.56 (0.54–0.58)	**< 0.001**
Integrated	0.71 (0.68–0.73)	**< 0.001**	0.77 (0.73–0.80)	**< 0.001**
**Cancer Type**				
Breast	—		—	
Colorectal	2.05 (1.76–2.38)	**< 0.001**	1.93 (1.64–2.26)	**< 0.001**
Endometrial	1.77 (1.35–2.30)	**< 0.001**	1.37 (1.03–1.81)	**0.028**
Kidney/Bladder	2.82 (2.52–3.16)	**< 0.001**	2.76 (2.44–3.12)	**< 0.001**
Liver	1.57 (0.94–2.53)	0.072	1.64 (0.96–2.71)	0.060
Lung	1.17 (1.07–1.29)	**< 0.001**	1.37 (1.24–1.50)	**< 0.001**
Lymphoma	0.21 (0.13–0.31)	**< 0.001**	0.20 (0.13–0.30)	**< 0.001**
Melanoma	2.72 (2.44–3.03)	**< 0.001**	2.76 (2.46–3.10)	**< 0.001**
Oral Cavity	0.69 (0.28–1.47)	0.378	0.51 (0.20–1.15)	0.13
Pancreas	1.14 (0.85–1.52)	0.37	1.22 (0.89–1.65)	0.21
Prostate	1.11 (0.77–1.58)	0.567	1.06 (0.72–1.52)	0.77
Thyroid	2.95 (1.91–4.53)	**< 0.001**	2.17 (1.36–3.46)	**0.001**
**Chemotherapy**				
No	—		—	
Yes	1.03 (1.00–1.06)	0.063	1.17 (1.13–1.21)	**< 0.001**
**Surgery Status**				
No Surgery Performed	—		—	
Surgery Performed	2.29 (2.17–2.43)	**< 0.001**	2.25 (2.11–2.40)	**< 0.001**
Race * Year Diagnosis				
American Indian or Alaskan Native * 2012–2020	—	—	0.92 (0.92–1.10)	0.83
Black * 2012–2020	—	—	1.12 (0.98–1.27)	0.091
East Asian * 2012–2020	—	—	1.10 (0.76–1.63)	0.62
Native Hawaiian or Pacific Islander * 2012–2020	—	—	0.57 (0.27–1.26)	0.15
South Asian * 2012–2020	—	—	0.75 (0.40–1.47)	0.39
Southeast Asian * 2012–2020	—	—	1.17 (0.76–1.88)	0.49

^*a*^*OR = Odds Ratio*,* CI = Confidence Interval*

^*b*^ *aOR = Adjusted Odds Ratio*

### Medicaid expansion analysis

In the Medicaid expansion sub-analysis, 45,721 patients (51%) met inclusion criteria as previously described in the Methods. Most patients had lung cancer as the primary diagnosis (85%), were White (84%), had a higher income (54%), had higher educational attainment (52%), underwent chemotherapy prior to RT (69%), did not have surgery prior to RT (93%), and had a comorbidity score of 0 (70%) (Table 3). Additionally, most patients were privately insured (58%), diagnosed in the pre-expansion period (51%), and lived in an expansion state (73%). Difference-in-difference analysis did not demonstrate a statistically significant difference in SRS versus WBRT utilization by Medicaid expansion status (aOR = 0.99; 95%CI = 0.88–1.10) (Table 4). Characteristics of the Medicaid expansion sub-analysis cohort by race are listed in Appendix 3.

**Table 3:** Characteristics of patients in medicaid expansion analysis

Characteristic	Overall (*N* = 45,721)^a^	WBRT^b^ (*N* = 33,993)	SRS^c^ (*N* = 11,728)	*P*-value
**Race, ***n* (%)				**< 0.001**
White	38,431 (84)	28,508 (84)	9,923 (85)	
American Indian or Alaskan Native	171 (0.4)	113 (0.3)	58 (0.5)	
Black	6,043 (13)	4,612 (14)	1,431 (12)	
East Asian	411 (0.9)	286 (0.8)	125 (1.1)	
Native Hawaiian or Pacific Islander	105 (0.2)	74 (0.2)	31 (0.3)	
South Asian	156 (0.3)	103 (0.3)	53 (0.5)	
Southeast Asian	404 (0.9)	297 (0.9)	107 (0.9)	
**Ethnicity**,* n ***(%)**				**0.005**
Non-Hispanic	44,105 (96)	32,840 (97)	11,265 (96)	
Hispanic	1,616 (3.5)	1,153 (3.4)	463 (3.9)	
**Sex**,* n*** (%)**				0.5
Male	22,999 (50)	17,067 (50)	5,932 (51)	
Female	22,722 (50)	16,926 (50)	5,796 (49)	
**Age**,** Median (IQR)**^**d**^	57 (53–61)	57 (52–61)	58 (53–61)	**< 0.001**
**Expansion Period**,* n*** (%)**				**< 0.001**
Pre-Expansion (2004–2013)	23,430 (51)	20,079 (59)	3,351 (29)	
Post-Expansion (2014–2020)	22,291 (49)	13,914 (41)	8,377 (71)	
**Medicaid Expansion Status**,* n*** (%)**				**< 0.001**
Non-Expansion	12,554 (27)	9,471 (28)	3,083 (26)	
Expansion	33,167 (73)	24,522 (72)	8,645 (74)	
**Distance To Hospital**,** Median (IQR)**	11 (5–24)	10 (4–22)	14 (6–31)	**< 0.001**
**Income**,* n*** (%)**				**< 0.001**
Higher Income	24,657 (54)	17,820 (52)	6,837 (58)	
Lower Income	21,064 (46)	16,173 (48)	4,891 (42)	
**Rurality**,* n*** (%)**				**< 0.001**
Metropolitan	37,519 (82)	27,747 (82)	9,772 (83)	
Urban-Rural	8,202 (18)	6,246 (18)	1,956 (17)	
**Education**,** n (%)**				**< 0.001**
More Education	23,560 (52)	17,032 (50)	6,528 (56)	
Less Education	22,161 (48)	16,961 (50)	5,200 (44)	
**Insurance Status**,* n*** (%)**				**< 0.001**
Private Insurance	26,436 (58)	18,979 (56)	7,457 (64)	
Medicaid/Medicare	15,481 (34)	11,769 (35)	3,712 (32)	
Uninsured	3,804 (8.3)	3,245 (9.5)	559 (4.8)	
**Comorbidity Index**,* n*** (%)**				**< 0.001**
0	32,138 (70)	23,668 (70)	8,470 (72)	
1	9,354 (20)	7,173 (21)	2,181 (19)	
2	2,834 (6.2)	2,116 (6.2)	718 (6.1)	
3+	1,395 (3.1)	1,036 (3.0)	359 (3.1)	
**US Region**,* n*** (%)**				**< 0.001**
Northeast	9,355 (20)	6,632 (20)	2,723 (23)	
Midwest	13,472 (29)	10,305 (30)	3,167 (27)	
South	16,647 (36)	12,463 (37)	4,184 (36)	
West	6,247 (14)	4,593 (14)	1,654 (14)	
**Facility Type**,* n*** (%)**				**< 0.001**
Academic	16,383 (36)	10,975 (32)	5,408 (46)	
Community	3,095 (6.8)	2,713 (8.0)	382 (3.3)	
Comprehensive Community	17,397 (38)	13,825 (41)	3,572 (30)	
Integrated	8,846 (19)	6,480 (19)	2,366 (20)	
**Cancer Type**,* n*** (%)**				**< 0.001**
Breast	1,811 (4.0)	1,423 (4.2)	388 (3.3)	
Colorectal	567 (1.2)	360 (1.1)	207 (1.8)	
Endometrial	167 (0.4)	121 (0.4)	46 (0.4)	
Kidney/Bladder	1,783 (3.9)	987 (2.9)	796 (6.8)	
Liver	39 (< 0.1)	29 (< 0.1)	< 11^e^ (< 0.1)	
Lung	38,715 (85)	29,429 (87)	9,286 (79)	
Lymphoma	188 (0.4)	182 (0.5)	< 11^e^ (< 0.1)	
Melanoma	2,185 (4.8)	1,263 (3.7)	922 (7.9)	
Oral Cavity	23 (< 0.1)	19 (< 0.1)	< 11^e^ (< 0.1)	
Pancreas	132 (0.3)	102 (0.3)	30 (0.3)	
Prostate	74 (0.2)	55 (0.2)	19 (0.2)	
Thyroid	37 (< 0.1)	23 (< 0.1)	14 (0.1)	
**Chemotherapy**,* n*** (%)**	31,774 (69)	23,585 (69)	8,189 (70)	0.4
**Surgery Status**,** n (%)**				**< 0.001**
No Surgery Performed	42,677 (93)	32,260 (95)	10,417 (89)	
Surgery Performed	3,044 (6.7)	1,733 (5.1)	1,311 (11)	

^*a*^ *Included only patients aged 40–64. Medicaid expansion status was unavailable for patients younger than 40 years and those 65 or older are eligible for Medicare*

^*b*^ *WBRT = Whole Brain Radiation Therapy*

^*c*^ *SRS = Stereotactic Radiosurgery*

^*d*^ *IQR = Interquartile Range*

^*e*^ *n < 11 masked per NCDB data privacy policy*

**Table 4:** Predictors of stereotactic radiosurgery utilization by medicaid expansion status

Characteristic	aOR (95%CI)^a^	*P*-value
**Race**		
White	—	
American Indian or Alaskan Native	1.50 (1.05–2.11)	**0.023**
Black	1.00 (0.93–1.07)	0.99
East Asian	1.11 (0.88–1.39)	0.28
Native Hawaiian or Pacific Islander	1.10 (0.69–1.72)	0.68
South Asian	1.29 (0.89–1.83)	0.17
Southeast Asian	0.95 (0.74–1.20)	0.65
**Expansion Period**		
Pre-Expansion (2004–2013)	—	
Post-Expansion (2014–2020)	3.87 (3.52–4.24)	**< 0.001**
**Medicaid Expansion Status**		
Non-Expansion	—	
Expansion	1.08 (0.98–1.19)	0.11
**Age**	1.00 (1.00–1.00)	0.90
**Distance To Hospital**	1.00 (1.00–1.00)	**< 0.001**
**Income**		
Higher Income	—	
Lower Income	0.90 (0.85–0.95)	**< 0.001**
**Rurality**		
Metropolitan	—	
Urban-Rural	1.01 (0.95–1.08)	0.68
**Education**		
More Education	—	
Less Education	0.87 (0.83–0.92)	**< 0.001**
**Insurance Status**		
Private Insurance	—	
Medicaid/Medicare	0.78 (0.75–0.83)	**< 0.001**
Uninsured	0.50 (0.46–0.56)	**< 0.001**
**Comorbidity Index**		
0	—	
1	0.92 (0.87–0.97)	**0.004**
2	1.00 (0.91–1.10)	0.94
3+	0.95 (0.83–1.08)	0.43
**US Region**		
Northeast	—	
Midwest	0.79 (0.74–0.84)	**< 0.001**
South	0.97 (0.89–1.05)	0.40
West	0.95 (0.87–1.02)	0.17
**Facility Type**		
Academic	—	
Community	0.30 (0.27–0.34)	**< 0.001**
Comprehensive Community	0.54 (0.51–0.57)	**< 0.001**
Integrated	0.79 (0.74–0.84)	**< 0.001**
**Cancer Type**		
Breast	—	
Colorectal	1.95 (1.56–2.42)	**< 0.001**
Endometrial	1.14 (0.77–1.66)	0.49
Kidney/Bladder	2.92 (2.49–3.44)	**< 0.001**
Liver	1.48 (0.66–3.08)	0.32
Lung	1.44 (1.27–1.63)	**< 0.001**
Lymphoma	0.14 (0.05–0.29)	**< 0.001**
Melanoma	2.90 (2.49–3.39)	**< 0.001**
Oral Cavity	1.01 (0.28–2.86)	0.99
Pancreas	1.19 (0.75–1.84)	0.45
Prostate	1.11 (0.62–1.92)	0.71
Thyroid	1.75 (0.83–3.56)	0.13
**Chemotherapy**		
No	—	
Yes	1.17 (1.11–1.23)	**< 0.001**
**Surgery Status**		
No Surgery Performed	—	
Surgery Performed	2.26 (2.08–2.47)	**< 0.001**
**Sex**		
Male	—	
Female	1.04 (1.00 − 1.09)	0.073
**Ethnicity**		
Non-Hispanic	—	
Hispanic	1.09 (0.96–1.23)	0.18
Expansion Period * Medicaid Expansion Status		
Post-Expansion (2014–2020) * Expansion	0.99 (0.88–1.10)	0.79

^*a*^ *OR = Odds Ratio*,* CI = Confidence Interval*

## Discussion

### Principal findings

In this large, national hospital-representative cohort study of more than 89,000 patients with BM from the 12 most prevalent cancers treated between 2004 and 2020, we identified a significant increase in the utilization of SRS. Despite SRS use rising from 8 to 54% over the study period, disparities persist across multiple sociodemographic, geographic, and institutional factors. Patients with lower socioeconomic status, no insurance or covered by Medicare/Medicare, and those treated at non-academic medical centers were less likely to receive SRS. These findings highlight persistent inequities in treatment patterns, though the extent to which these reflect access barriers versus differences in clinical presentation (i.e., number of BMs, extent of intracranial disease) cannot be determined from our data. Compared to WBRT, SRS offers the potential for reduced neurocognitive toxicity and is preferred for patients with limited BM, particularly those with good performance status and fewer lesions [21]. These benefits (i.e., preserving quality of life, memory, and cognition) underscore the need to understand whether these disparities reflect inequitable access to advanced RT options or appropriate clinical decision-making based on disease characteristics not captured in our dataset [22, 23].

Our findings build upon prior research documenting disparities in the use of SRS versus WBRT for BM management [7, 8]. Expanding on the work of Kann et al., we extended the analysis to more recent years, a broader range of primary cancer sites, disaggregated racial and ethnic groups, and the effects of Medicaid expansion. We found that South Asian patients were more likely to receive SRS. While based on a small sample size within the South Asian subgroup, this association underscores how disaggregated data can reveal practice patterns masked by broader racial categories. Race and ethnicity were otherwise not independently associated with SRS utilization, suggesting that disparities affecting Black and other non-White patients identified in previous studies may have improved, or are at least not being exacerbated [7]. These findings may reflect gradual improvements in access, potentially driven by shifting referral patterns, reduced implicit bias, or improved insurance coverage. However, given the absence of a positive association and the lack of change in SRS utilization by racial groups over time (as shown in the interaction term analysis), these findings warrant cautious interpretation and highlight the need for further study to evaluate this trend. Provider bias, referral patterns, and patient preferences may still influence access and require further investigation with real-world studies to explore contributing factors, stratified by cancer type, socioeconomic status, and treatment facility characteristics.

We also found that females were more likely than males to receive SRS. While the prior study by Kann et al. did not identify sex as a factor associated with SRS use in BM, our findings align with existing literature showing that female sex is an independent predictor of improved survival following SRS [24–26]. These survival differences may lead clinicians to preferentially offer SRS to female patients, particularly if they are perceived to have a more favorable prognosis. Female patients often exhibit more favorable tumor characteristics, such as smaller lesion sizes and differing primary cancer distributions, with hormone receptor–positive breast cancer comprising a substantial proportion of BM in women [27]. Additionally, a previous NCDB study in breast cancer patients found that women were more likely to refuse surgery and more likely to accept radiation therapy compared to men [28]. These differences may also reflect broader patterns in care shaped by provider assumptions, referral practices, or other unmeasured clinical or social factors. However, few studies have directly examined sex-based differences in SRS utilization, and further research is needed to determine whether these patterns reflect appropriate clinical decision-making or inequities in treatment delivery that could affect male patients with similar clinical profiles.

In contrast to prior studies, we did not identify Hispanic ethnicity as a predictor of lower SRS use [7, 8]. This may reflect the results of ongoing efforts and advocacy for improving access to cancer care in the United States, such as the federally mandated expansion of community outreach and engagement (COE) in 2016 [29]. Alternatively, differences in cohort composition such as sample size, geographic representation, and primary cancer site inclusion may explain this discrepancy, as our time interaction analysis showed no significant temporal trends among Hispanic patients. Further research is needed to determine whether this trend reflects true improvements in access to advanced RT or is influenced by underlying demographic and institutional factors.

### Possible impact of medicaid expansion on SRS practice patterns

To our knowledge, this study is among the first to evaluate the impact of Medicaid expansion on SRS utilization for BM. Contrary to expectations, we found no significant differences in SRS use between Medicaid expansion and non-expansion states. This may be partly due to the lower availability of SRS and limited MRI capacity at centers serving higher proportions of Medicaid populations, as well as the composition of hospitals that participate in the NCDB. The NCDB primarily includes data from Commission on Cancer-accredited hospitals, which may not fully capture patterns of care in non-accredited facilities that serve more Medicaid-insured patients [30]. While Medicaid expansion has improved overall healthcare utilization and increased insurance coverage for cancer patients, access gaps persist, particularly for high-cost, specialized treatments such as SRS [31–33]. These findings challenge the assumption that expanded insurance coverage alone can eliminate disparities in access to advanced cancer therapies. Factors such as geographic access, availability of specialized RT centers, referral patterns, and institutional resources likely play more decisive roles in shaping treatment utilization among Medicaid beneficiaries. Notably, we found no significant changes in these disparities over time, further emphasizing the need for targeted efforts to address structural barriers in community and non-academic settings.

### Socioeconomic and geographic disparities

Socioeconomic and geographic disparities in SRS utilization reflect broader inequities in access to advanced cancer treatments. Patients with lower income, less educational attainment, no insurance, or Medicare/Medicaid coverage were significantly less likely to receive SRS, even after adjusting for clinical factors. The rise of managed Medicare and Medicaid plans may further complicate access to specialized technologies like SRS due to high costs and complex approvals processes [8, 34]. Thus, cost and access remain major barriers to treatment of BM, particularly for newer, more precise therapies like SRS, despite its reduced neurocognitive toxicity compared to conventional approaches [35]. Regional disparities were also evident, with patients in the Midwest being less likely to receive SRS compared to those in the Northeast, potentially due to differences in the availability of advanced radiation technologies and expertise [36]. This variation highlights the influence of institutional factors, such as the presence of academic medical centers and specialized oncology services, in treatment decisions. Future studies should investigate these institutional practices and develop strategies to improve access to specialized care in under-resourced regions [37].

In addition to disparities in access, these observed patterns may also be due to differences in disease severity at presentation amongst certain populations. Previous research suggests that patients from lower socioeconomic backgrounds may present with more advanced intracranial disease due to delayed healthcare seeking, poor health literacy, or financial concerns about medical costs [38–40]. Studies have also consistently demonstrated that both uninsured patients and those with lower socioeconomic status are significantly more likely to present with more advanced cancer at diagnosis across multiple cancer types [41–43]. Simultaneously, geographic barriers to accessing specialized centers with SRS capabilities may limit treatment options even for patients who would otherwise be appropriate SRS candidates. Our findings likely reflect practice patterns due to a complex interplay of factors. These include disparate disease burden that appropriately guides treatment selection and genuine barriers to accessing advanced RT technologies, with the relative contribution of each factor varying by region and patient population.

### Policy implications

Our findings have important implications for healthcare policy aimed at increasing access to treatment for all cancer patients. Clinicians and policymakers should collaborate to expand insurance coverage, enhance access to advanced radiation therapy, and address structural and institutional barriers to equitable care [37, 44]. The limited impact of Medicaid expansion on SRS utilization may reflect that patients with Stage IV cancer likely have already qualified for Medicaid prior to the Affordable Care Act, reducing the policy’s measurable impact in this population. Additionally, disparities may stem more from challenges in obtaining and utilizing expanded insurance coverage than from eligibility alone [45]. Future policies should prioritize technology diffusion to ensure equitable access to advanced, particularly in under-resourced regions. Efforts should also address structural barriers that limit access to SRS, such as limited specialized equipment and institutional capacity. Federal initiatives, like the Radiation Oncology Case Rate Value-Based Payment Program, could help standardize SRS reimbursement, equalizing payment structures and promoting equitable treatment practices [46].

Targeted efforts are necessary to address structural, financial, and geographic factors contributing to persistent disparities observed in our study. To mitigate disparities associated with more advanced disease presentation among patients of lower socioeconomic status or those uninsured or publicly insured, which may render SRS clinically inappropriate due to extensive intracranial disease burden, policies should prioritize community-based initiatives such as expanded cancer screening, patient navigation programs, and culturally tailored education campaigns aimed at improving health literacy and promoting earlier diagnosis. Concurrently, institutional and geographic barriers to accessing advanced RT modalities could be alleviated by targeted financial investments or subsidies directed toward non-academic and community-based facilities to enhance infrastructure, technology acquisition, and personnel training in advanced RT techniques, including SRS. Additionally, implementing equitable reimbursement policies, such as value-based payment models specifically incentivizing the use of advanced RT modalities, and expanding telemedicine resources to facilitate specialty consultation could further reduce geographic disparities. Addressing the persistent disparities in SRS utilization will require a multifaceted approach that addresses prevention, access, and infrastructure considerations across different socioeconomic and geographic contexts.

### Limitations

This study has limitations. First, the NCDB is restricted to patients treated at Commission on Cancer-accredited facilities, which may limit generalizability, particularly in regions with fewer accredited centers where SRS may be underreported. Additionally, the NCDB captures only first-course treatments, preventing analysis of patients who received both WBRT and SRS in sequence. The database also lacks information on the number of BM and the extent of intracranial disease, critical factors influencing treatment decisions and clinical outcomes [47]. These gaps limit our ability to assess whether the preference for SRS versus WBRT aligns with current guidelines, which prioritize SRS for patients with limited BM or cases with good performance status [19]. As such, our findings may not fully capture the nuances of clinical decision-making based on disease burden. The lack of linkage across treatment centers also prevents tracking of patients who underwent multiple rounds of radiation therapy. Observed trends may also be impacted by increased use of central nervous system-active systemic therapeutics in recent years, which was not evaluated in this analysis. Additionally, the pooled analysis of Medicare and Medicaid patients may have masked differences in SRS utilization between Medicaid expansion and non-expansion states. The NCDB also lacks data on Medicaid expansion status for patients younger than 40, limiting the generalizability of our findings to younger populations. The retrospective design also limits insight into factors influencing treatment choices and their impact on outcomes such as tolerability, morbidity, and quality of life. Finally, the study does not account for potential changes in treatment patterns due to the COVID-19 pandemic, as data extend only through 2020. Future research should address these gaps to better understand disparities in SRS access and utilization.

## Conclusions

In one of the largest United States studies of BM including over 89,000 patients with BM treated from 2004 to 2020, we identified persistent disparities in the utilization of SRS. Our analysis echoes prior studies while providing a unique contribution by examining a broad, contemporary cohort spanning nearly two decades, capturing trends across major policy changes like Medicaid expansion. These findings are significant, as SRS offers superior precision, reduced neurocognitive toxicity, and better quality-of-life outcomes compared to WBRT, particularly for patients with limited BM. Despite increased adoption, disparities in access have not improved with Medicaid expansion. Socioeconomic and geographic disparities in SRS use remain evident, suggesting that insurance expansion alone may be insufficient to ensure equitable access. As SRS becomes more widely adopted with an expanding set of therapeutic indications, further research is needed to distinguish whether these patterns reflect barriers to access or disparities in severity of intracranial disease upon presentation that influence chosen treatment modality. If access barriers are confirmed as primary drivers, targeted interventions may be needed to address structural, financial, and geographic factors that influence SRS adoption and support equitable access.

## Electronic supplementary material

Below is the link to the electronic supplementary material.

Supplementary Material 1

Supplementary Material 2

Supplementary Material 3

## Data Availability

Research data from the National Cancer Database are available upon request from the American Cancer Society and the American College of Surgeons (http://ncdbpuf.facs.org/).
