# Supplementary figures and images for: Stereotactic radiosurgery for brain metastases: evolving practice patterns from the national cancer database (2004–2020)
Source: J Neurooncol. 2025 Aug 22;175(3):1211–25. doi: 10.1007/s11060-025-05178-8 (PMC12511135; doi:10.1007/s11060-025-05178-8)

**Appendix 1.** Inclusion and Exclusion Criteria for Study Cohort
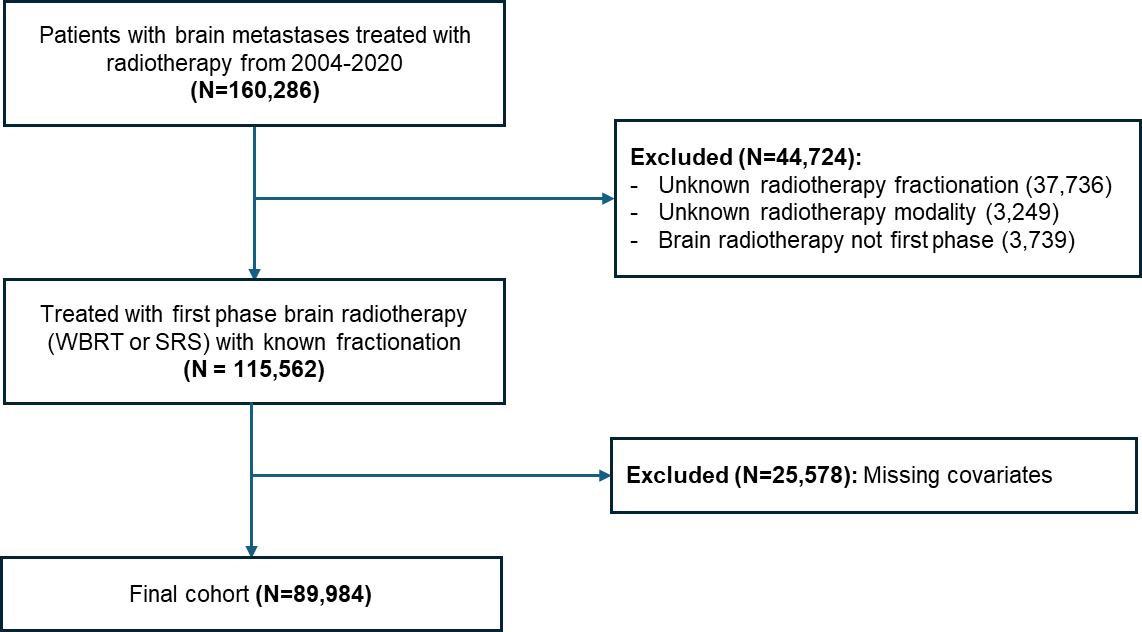

Supplement: Supplementary file 1 — Supplementary Material 1 [file 11060_2025_5178_MOESM1_ESM.docx]
