# Supplementary material for: Stereotactic radiosurgery for brain metastases: evolving practice patterns from the national cancer database (2004–2020)
Source: J Neurooncol. 2025 Aug 22;175(3):1211–25. doi: 10.1007/s11060-025-05178-8 (PMC12511135; doi:10.1007/s11060-025-05178-8)
Supplement: Supplementary file 2 — Supplementary Material 2 [file 11060_2025_5178_MOESM2_ESM.docx]

| **Appendix 2. Characteristics by Race of Patients with Brain Metastases Treated with Radiotherapy from 2004 to 2020** | | | | | | | | | |
| --- | --- | --- | --- | --- | --- | --- | --- | --- | --- |
| **Characteristic** | **Overall**  **(N = 89,984)** | **White**  **(N = 77,556)** | **AIAN^a^**  **(N = 296)** | **Black**  **(N = 10,028)** | **East Asian**  **(N=912)** | **NHPI^b^**  **(N=168)** | **South Asian**  **(N=250)** | **Southeast Asian**  **(N=774)** | **P-value** |
| **Ethnicity, n (%)** |  |  |  |  |  |  |  |  | <0.001 |
| Non-Hispanic | 87,186 (97) | 74,886 (97) | 289 (98) | 9,925 (99) | 910 (100) | 164 (98) | 249 (100) | 763 (99) |  |
| Hispanic | 2,798 (3.1) | 2,670 (3.4) | <11^g^ (2.4) | 103 (1.0) | <11^g^ (0.2) | <11^g^ (2.4) | <11^g^ (0.4) | 11 (1.4) |  |
| **RT^c^ Modality, n (%)** |  |  |  |  |  |  |  |  | <0.001 |
| WBRT^d^ | 65,810 (73) | 56,646 (73) | 209 (71) | 7,498 (75) | 632 (69) | 115 (68) | 158 (63) | 552 (71) |  |
| SRS^e^ | 24,174 (27) | 20,910 (27) | 87 (29) | 2,530 (25) | 280 (31) | 53 (32) | 92 (37) | 222 (29) |  |
| **Sex, n (%)** |  |  |  |  |  |  |  |  | 0.003 |
| Male | 46,296 (51) | 39,978 (52) | 128 (43) | 5,068 (51) | 479 (53) | 84 (50) | 150 (60) | 409 (53) |  |
| Female | 43,688 (49) | 37,578 (48) | 168 (57) | 4,960 (49) | 433 (47) | 84 (50) | 100 (40) | 365 (47) |  |
| **Age, Median (IQR)^f^** | 64 (57–72) | 65 (58–72) | 63 (56–69) | 62 (55–69) | 66 (58–74) | 60 (54–69) | 62 (55–69) | 64 (56–71) | <0.001 |
| **Year Diagnosis, n (%)** |  |  |  |  |  |  |  |  | <0.001 |
| 2004-2011 | 31,975 (36) | 27,785 (36) | 65 (22) | 3,471 (35) | 295 (32) | 56 (33) | 71 (28) | 232 (30) |  |
| 2012-2020 | 58,009 (64) | 49,771 (64) | 231 (78) | 6,557 (65) | 617 (68) | 112 (67) | 179 (72) | 542 (70) |  |
| **Distance To Hospital, Median (IQR)** | 10 (4–23) | 11 (5–25) | 23 (6–58) | 6 (3–13) | 6 (3–11) | 8 (4–18) | 8 (4–13) | 7 (3–12) | <0.001 |
| **Income, n (%)** |  |  |  |  |  |  |  |  | <0.001 |
| Higher Income | 50,860 (57) | 46,108 (59) | 95 (32) | 3,011 (30) | 714 (78) | 128 (76) | 202 (81) | 602 (78) |  |
| Lower Income | 39,124 (43) | 31,448 (41) | 201 (68) | 7,017 (70) | 198 (22) | 40 (24) | 48 (19) | 172 (22) |  |
| **Rurality, n (%)** |  |  |  |  |  |  |  |  | <0.001 |
| Metropolitan | 74,092 (82) | 62,848 (81) | 155 (52) | 9,053 (90) | 893 (98) | 150 (89) | 248 (99) | 745 (96) |  |
| Urban-Rural | 15,892 (18) | 14,708 (19) | 141 (48) | 975 (9.7) | 19 (2.1) | 18 (11) | 2 (0.8) | 29 (3.7) |  |
| **Education, n (%)** |  |  |  |  |  |  |  |  | <0.001 |
| More Education | 49,116 (55) | 45,225 (58) | 111 (38) | 2,663 (27) | 501 (55) | 104 (62) | 156 (62) | 356 (46) |  |
| Less Education | 40,868 (45) | 32,331 (42) | 185 (63) | 7,365 (73) | 411 (45) | 64 (38) | 94 (38) | 418 (54) |  |
| **Insurance Status, n (%)** |  |  |  |  |  |  |  |  | <0.001 |
| Private Insurance | 31,713 (35) | 27,910 (36) | 89 (30) | 2,896 (29) | 321 (35) | 64 (38) | 112 (45) | 321 (41) |  |
| Medicaid/Medicare | 54,106 (60) | 46,332 (60) | 195 (66) | 6,399 (64) | 553 (61) | 99 (59) | 114 (46) | 414 (53) |  |
| Uninsured | 4,165 (4.6) | 3,314 (4.3) | 12 (4.1) | 733 (7.3) | 38 (4.2) | <11^g^ (3.0) | 24 (9.6) | 39 (5.0) |  |
| **Comorbidity Index, n (%)** |  |  |  |  |  |  |  |  | <0.001 |
| 0 | 59,409 (66) | 51,175 (66) | 183 (62) | 6,471 (65) | 702 (77) | 117 (70) | 172 (69) | 589 (76) |  |
| 1 | 20,008 (22) | 17,360 (22) | 74 (25) | 2,207 (22) | 154 (17) | 34 (20) | 56 (22) | 123 (16) |  |
| 2 | 6,916 (7.7) | 5,987 (7.7) | 25 (8.4) | 808 (8.1) | 38 (4.2) | <11^g^ (5.4) | 15 (6.0) | 34 (4.4) |  |
| 3+ | 3,651 (4.1) | 3,034 (3.9) | 14 (4.7) | 542 (5.4) | 18 (2.0) | <11^g^ (4.8) | <11^g^ (2.8) | 28 (3.6) |  |
| **US Region, n (%)** |  |  |  |  |  |  |  |  | <0.001 |
| Northeast | 19,022 (21) | 16,849 (22) | 24 (8.1) | 1,721 (17) | 226 (25) | 16 (9.5) | 89 (36) | 97 (13) |  |
| Midwest | 26,326 (29) | 23,544 (30) | 97 (33) | 2,519 (25) | 50 (5.5) | <11^g^ (4.2) | 41 (16) | 68 (8.8) |  |
| South | 31,492 (35) | 25,855 (33) | 96 (32) | 5,191 (52) | 104 (11) | 16 (9.5) | 77 (31) | 153 (20) |  |
| West | 13,144 (15) | 11,308 (15) | 79 (27) | 597 (6.0) | 532 (58) | 129 (77) | 43 (17) | 456 (59) |  |
| **Facility Type, n (%)** |  |  |  |  |  |  |  |  | <0.001 |
| Academic | 29,943 (33) | 24,288 (31) | 73 (25) | 4,613 (46) | 462 (51) | 77 (46) | 128 (51) | 302 (39) |  |
| Community | 6,452 (7.2) | 5,790 (7.5) | 49 (17) | 477 (4.8) | 43 (4.7) | 14 (8.3) | 13 (5.2) | 66 (8.5) |  |
| Comp. Community | 35,843 (40) | 32,070 (41) | 135 (46) | 3,032 (30) | 258 (28) | 42 (25) | 62 (25) | 244 (32) |  |
| Integrated | 17,746 (20) | 15,408 (20) | 39 (13) | 1,906 (19) | 149 (16) | 35 (21) | 47 (19) | 162 (21) |  |
| **Cancer Type, n (%)** |  |  |  |  |  |  |  |  | —^h^ |
| Breast | 2,803 (3.1) | 2,206 (2.8) | 12 (4.1) | 515 (5.1) | 26 (2.9) | <11^g^ (4.2) | 18 (7.2) | 19 (2.5) |  |
| Colorectal | 1,100 (1.2) | 928 (1.2) | <11^g^ (3.0) | 147 (1.5) | 10 (1.1) | <11^g^ (0.6) | <11^g^ (0.4) | <11^g^ (0.5) |  |
| Endometrial | 272 (0.3) | 232 (0.3) | <11^g^ (0.3) | 31 (0.3) | <11^g^ (0.1) | <11^g^ (2.4) | 0 (0) | <11^g^ (0.4) |  |
| Kidney/Bladder | 3,100 (3.4) | 2,803 (3.6) | 22 (7.4) | 220 (2.2) | 25 (2.7) | <11^g^ (2.4) | 13 (5.2) | 13 (1.7) |  |
| Liver | 77 (<0.1) | 61 (<0.1) | <11^g^ (0.3) | <11^g^ (<0.1) | <11^g^ (0.4) | 0 (0) | 0 (0) | <<11^g^ (0.1) |  |
| Lung | 77,406 (86) | 66,316 (86) | 239 (81) | 8,935 (89) | 832 (91) | 149 (89) | 215 (86) | 720 (93) |  |
| Lymphoma | 426 (0.5) | 358 (0.5) | <11^g^ (1.4) | 51 (0.5) | <11^g^ (0.4) | 0 (0) | 2 (0.8) | <11^g^ (0.9) |  |
| Melanoma | 4,232 (4.7) | 4,181 (5.4) | <11^g^ (2.0) | 36 (0.4) | <11^g^ (0.4) | <11^g^ (1.2) | 0 (0) | <11^g^ (0.4) |  |
| Oral Cavity | 42 (<0.1) | 37 (<0.1) | 0 (0) | <11^g^ (<0.1) | 0 (0) | 0 (0) | 0 (0) | 0 (0) |  |
| Pancreas | 266 (0.3) | 224 (0.3) | <11^g^ (0.3) | 35 (0.3) | <11^g^ (0.3) | 0 (0) | <11^f^ (0.4) | <11^g^ (0.3) |  |
| Prostate | 173 (0.2) | 141 (0.2) | 0 (0) | 30 (0.3) | <11^g^ (0.1) | <11^g^ (0.6) | 0 (0) | 0 (0) |  |
| Thyroid | 87 (<0.1) | 69 (<0.1) | <11^g^ (0.3) | 13 (0.1) | <11^g^ (0.2) | 0 (0) | 0 (0) | <11^g^ (0.3) |  |
| **Chemotherapy, n (%)** | 56,713 (63) | 48,924 (63) | 190 (64) | 6,121 (61) | 656 (72) | 111 (66) | 173 (69) | 538 (70) | <0.001 |
| **Surgery Status, n (%)** |  |  |  |  |  |  |  |  | <0.001 |
| No Surgery Performed | 84,861 (94) | 73,015 (94) | 274 (93) | 9,582 (96) | 862 (95) | 157 (93) | 238 (95) | 733 (95) |  |
| Surgery Performed | 5,123 (5.7) | 4,541 (5.9) | 22 (7.4) | 446 (4.4) | 50 (5.5) | 11 (6.5) | 12 (4.8) | 41 (5.3) |  |
| ^a^AIAN = American Indian or Alaskan Native  ^b^NHPI = Native Hawaiian or Pacific Islander  ^c^RT = Radiotherapy  ^d^WBRT = *Whole Brain Radiation Therapy*  *^e^SRS = Stereotactic Radiosurgery*  *^f^IQR = Interquartile Range*  *^g^n <11 masked per NCDB data privacy policy*  ^h^P-value cannot be calculated due to small N | | | | | | | | | |
