# Supplementary material for: Stereotactic radiosurgery for brain metastases: evolving practice patterns from the national cancer database (2004–2020)
Source: J Neurooncol. 2025 Aug 22;175(3):1211–25. doi: 10.1007/s11060-025-05178-8 (PMC12511135; doi:10.1007/s11060-025-05178-8)
Supplement: Supplementary file 3 — Supplementary Material 3 [file 11060_2025_5178_MOESM3_ESM.docx]

| **Appendix 3. Characteristics by Race of Patients in Medicaid Expansion Analysis** | | | | | | | | | |
| --- | --- | --- | --- | --- | --- | --- | --- | --- | --- |
| **Characteristic** | **Overall**  **(N = 89,984)** | **White**  **(N = 77,556)** | **AIAN^a^**  **(N = 296)** | **Black**  **(N = 10,028)** | **East Asian**  **(N=912)** | **NHPI^b^**  **(N=168)** | **South Asian**  **(N=250)** | **Southeast Asian**  **(N=774)** | **P-value** |
| **Ethnicity, n (%)** |  |  |  |  |  |  |  |  | —^h^ |
| Non-Hispanic | 44,105 (96) | 36,895 (96) | 167 (98) | 5,979 (99) | 409 (100) | 101 (96) | 156 (100) | 398 (99) |  |
| Hispanic | 1,616 (3.5) | 1,536 (4.0) | <11^g^(2.3) | 64 (1.1) | <11^g^ (0.5) | <11^g^ (3.8) | 0 (0) | <11^g^ (1.5) |  |
| **RT^c^ Modality, n (%)** |  |  |  |  |  |  |  |  | <0.001 |
| WBRT^d^ | 33,993 (74) | 28,508 (74) | 113 (66) | 4,612 (76) | 286 (70) | 74 (70) | 103 (66) | 297 (74) |  |
| SRS^e^ | 11,728 (26) | 9,923 (26) | 58 (34) | 1,431 (24) | 125 (30) | 31 (30) | 53 (34) | 107 (26) |  |
| **Sex, n (%)** |  |  |  |  |  |  |  |  | 0.5 |
| Male | 22,999 (50) | 19,290 (50) | 76 (44) | 3,065 (51) | 220 (54) | 55 (52) | 82 (53) | 211 (52) |  |
| Female | 22,722 (50) | 19,141 (50) | 95 (56) | 2,978 (49) | 191 (46) | 50 (48) | 74 (47) | 193 (48) |  |
| **Age, Median (IQR)^f^** | 57 (53–61) | 57 (53–61) | 57 (52–61) | 57 (52–61) | 57 (52–61) | 55 (51–59) | 56 (51–61) | 57 (50– 61) | <0.001 |
| **Expansion Period, n (%)** |  |  |  |  |  |  |  |  | 0.004 |
| Pre-Expansion (2004- 2013) | 23,430 (51) | 19,790 (51) | 69 (40) | 3,059 (51) | 208 (51) | 49 (47) | 67 (43) | 188 (47) |  |
| Post-Expansion (2014-2020) | 22,291 (49) | 18,641 (49) | 102 (60) | 2,984 (49) | 203 (49) | 56 (53) | 89 (57) | 216 (53) |  |
| **Expansion Period, n (%)** |  |  |  |  |  |  |  |  | —^h^ |
| Non-Expansion | 12,554 (27) | 10,006 (26) | 54 (32) | 2,353 (39) | 26 (6.3) | 9 (8.6) | 34 (22) | 72 (18) |  |
| Expansion | 33,167 (73) | 28,425 (74) | 117 (68) | 3,690 (61) | 385 (94) | 96 (91) | 122 (78) | 332 (82) |  |
| **Distance To Hospital, Median (IQR)** | 11 (5–24) | 12 (5–26) | 24 (7–69) | 6 (3–13) | 6 (3–11) | 7 (4–17) | 8 (4–15) | 7 (3–13) | <0.001 |
| **Income, n (%)** |  |  |  |  |  |  |  |  | <0.001 |
| Higher Income | 24,657 (54) | 22,036 (57) | 53 (31) | 1,749 (29) | 317 (77) | 77 (73) | 124 (79) | 301 (75) |  |
| Lower Income | 21,064 (46) | 16,395 (43) | 118 (69) | 4,294 (71) | 94 (23) | 28 (27) | 32 (21) | 103 (25) |  |
| **Rurality, n (%)** |  |  |  |  |  |  |  |  | <0.001 |
| Metropolitan | 37,519 (82) | 30,961 (81) | 86 (50) | 5,428 (90) | 402 (98) | 96 (91) | 154 (99) | 392 (97) |  |
| Urban-Rural | 8,202 (18) | 7,470 (19) | 85 (50) | 615 (10) | <11^g^ (2.2) | <11^g^ (8.6) | <11^g^ (1.3) | 12 (3.0) |  |
| **Education, n (%)** |  |  |  |  |  |  |  |  | <0.001 |
| More Education | 23,560 (52) | 21,406 (56) | 66 (39) | 1,530 (25) | 218 (53) | 62 (59) | 92 (59) | 186 (46) |  |
| Less Education | 22,161 (48) | 17,025 (44) | 105 (61) | 4,513 (75) | 193 (47) | 43 (41) | 64 (41) | 218 (54) |  |
| **Insurance Status, n (%)** |  |  |  |  |  |  |  |  | <0.001 |
| Private Insurance | 26,436 (58) | 23,293 (61) | 67 (39) | 2,428 (40) | 245 (60) | 57 (54) | 89 (57) | 257 (64) |  |
| Medicaid/Medicare | 15,481 (34) | 12,112 (32) | 93 (54) | 2,930 (48) | 135 (33) | 43 (41) | 50 (32) | 118 (29) |  |
| Uninsured | 3,804 (8.3) | 3,026 (7.9) | 11 (6.4) | 685 (11) | 31 (7.5) | <11^g^ (4.8) | 17 (11) | 29 (7.2) |  |
| **Comorbidity Index, n (%)** |  |  |  |  |  |  |  |  | —^h^ |
| 0 | 32,138 (70) | 26,958 (70) | 112 (65) | 4,201 (70) | 351 (85) | 73 (70) | 113 (72) | 330 (82) |  |
| 1 | 9,354 (20) | 8,018 (21) | 38 (22) | 1,157 (19) | 44 (11) | 18 (17) | 31 (20) | 48 (12) |  |
| 2 | 2,834 (6.2) | 2,362 (6.1) | 14 (8.2) | 416 (6.9) | 11 (2.7) | <11^g^ (7.6) | <11^g^ (3.8) | 17 (4.2) |  |
| 3+ | 1,395 (3.1) | 1,093 (2.8) | <11^g^ (4.1) | 269 (4.5) | <11^g^ (1.2) | <11^g^ (5.7) | <11^g^ (3.8) | <11^g^ (2.2) |  |
| **US Region, n (%)** |  |  |  |  |  |  |  |  | <0.001 |
| Northeast | 9,355 (20) | 8,097 (21) | 16 (9.4) | 1,012 (17) | 102 (25) | <11^g^ (9.5) | 56 (36) | 62 (15) |  |
| Midwest | 13,472 (29) | 11,800 (31) | 60 (35) | 1,529 (25) | 25 (6.1) | <11^g^ (4.8) | 23 (15) | 30 (7.4) |  |
| South | 16,647 (36) | 13,253 (34) | 53 (31) | 3,129 (52) | 55 (13) | 12 (11) | 53 (34) | 92 (23) |  |
| West | 6,247 (14) | 5,281 (14) | 42 (25) | 373 (6.2) | 229 (56) | 78 (74) | 24 (15) | 220 (54) |  |
| **Facility Type, n (%)** |  |  |  |  |  |  |  |  | <0.001 |
| Academic | 16,383 (36) | 12,856 (33) | 49 (29) | 2,970 (49) | 227 (55) | 46 (44) | 78 (50) | 157 (39) |  |
| Community | 3,095 (6.8) | 2,722 (7.1) | 24 (14) | 285 (4.7) | <11^g^ (2.2) | 11 (10) | <11^g^ (6.4) | 34 (8.4) |  |
| Comp. Community | 17,397 (38) | 15,317 (40) | 68 (40) | 1,701 (28) | 110 (27) | 28 (27) | 41 (26) | 132 (33) |  |
| Integrated | 8,846 (19) | 7,536 (20) | 30 (18) | 1,087 (18) | 65 (16) | 20 (19) | 27 (17) | 81 (20) |  |
| **Cancer Type, n (%)** |  |  |  |  |  |  |  |  | —^h^ |
| Breast | 1,811 (4.0) | 1,378 (3.6) | <11^g^ (5.3) | 368 (6.1) | 18 (4.4) | <11^g^ (5.7) | 15 (9.6) | 17 (4.2) |  |
| Colorectal | 567 (1.2) | 459 (1.2) | <11^g^ (4.7) | 90 (1.5) | <11^g^ (1.0) | <11^g^ (1.0) | <11^g^ (0.6) | <11^g^ (1.0) |  |
| Endometrial | 167 (0.4) | 138 (0.4) | <11^g^ (0.6) | 20 (0.3) | <11^g^ (0.2) | <11^g^ (3.8) | 0 (0) | <11^g^ (0.7) |  |
| Kidney/Bladder | 1,783 (3.9) | 1,602 (4.2) | 18 (11) | 133 (2.2) | 11 (2.7) | <11^g^ (1.0) | <11^g^ (5.8) | <11^g^ (2.2) |  |
| Liver | 39 (<0.1) | 30 (<0.1) | 0 (0) | <11^g^ (<0.1) | <11^g^ (1.0) | 0 (0) | 0 (0) | <11^g^ (0.2) |  |
| Lung | 38,715 (85) | 32,315 (84) | 129 (75) | 5,322 (88) | 367 (89) | 92 (88) | 130 (83) | 360 (89) |  |
| Lymphoma | 188 (0.4) | 146 (0.4) | <11^g^ (0.6) | 35 (0.6) | <11^g^ (0.2) | 0 (0) | <11^g^ (0.6) | <11^g^ (1.0) |  |
| Melanoma | 2,185 (4.8) | 2,150 (5.6) | <11^g^ (1.8) | 26 (0.4) | <11^g^ (0.7) | <11^g^ (1.0) | 0 (0) | <11^g^ (0.5) |  |
| Oral Cavity | 23 (<0.1) | 21 (<0.1) | 0 (0) | <11^g^ (<0.1) | 0 (0) | 0 (0) | 0 (0) | 0 (0) |  |
| Pancreas | 132 (0.3) | 109 (0.3) | <11^g^ (0.6) | 19 (0.3) | <11^g^ (0.2) | 0 (0) | 0 (0) | <11^g^ (0.5) |  |
| Prostate | 74 (0.2) | 56 (0.1) | 0 (0) | 18 (0.3) | 0 (0) | 0 (0) | 0 (0) | 0 (0) |  |
| Thyroid | 37 (<0.1) | 27 (<0.1) | <11^g^ (0.6) | <11^g^ (<0.1) | <11^g^ (0.2) | 0 (0) | 0 (0) | <11^g^ (0.5) |  |
| **Chemotherapy, n (%)** | 31,774 (69) | 26,882 (70) | 119 (70) | 3,965 (66) | 312 (76) | 71 (68) | 120 (77) | 305 (75) | <0.001 |
| **Surgery Status, n (%)** |  |  |  |  |  |  |  |  | <0.001 |
| No Surgery Performed | 42,677 (93) | 35,786 (93) | 154 (90) | 5,733 (95) | 385 (94) | 99 (94) | 146 (94) | 374 (93) |  |
| Surgery Performed | 3,044 (6.7) | 2,645 (6.9) | 17 (9.9) | 310 (5.1) | 26 (6.3) | <11^g^ (5.7) | <11^g^ (6.4) | 30 (7.4) |  |
| ^a^AIAN = American Indian or Alaskan Native  ^b^NHPI = Native Hawaiian or Pacific Islander  ^c^RT = Radiotherapy  ^d^WBRT = *Whole Brain Radiation Therapy*  *^e^SRS = Stereotactic Radiosurgery*  *^f^IQR = Interquartile Range*  *^g^n <11 masked per NCDB data privacy policy*  ^h^P-value cannot be calculated due to small N | | | | | | | | | |
